# Supplementary material for: Testing the Resilience, Physiological Plasticity and Mechanisms Underlying Upper Temperature Limits of Antarctic Marine Ectotherms
Source: Biology (Basel). 2024 Mar 29;13(4):224. doi: 10.3390/biology13040224 (PMC11047991; doi:10.3390/biology13040224)
Supplement: Supplementary file 1 [file biology-13-00224-s001.zip › biology-2924231-supplementary.pdf]

Article

Testing the resilience, physiological plasticity and mechanisms underlying upper temperature limits of Antarctic marine ectotherms

Simon A. Morley[1], Amanda E. Bates[2], Melody S. Clark[1], Elaine Fitzcharles[1], Rebecca Smith[1], Rose E. Stainthorp[1,3], Lloyd S. Peck[1]

Supplementary Materials

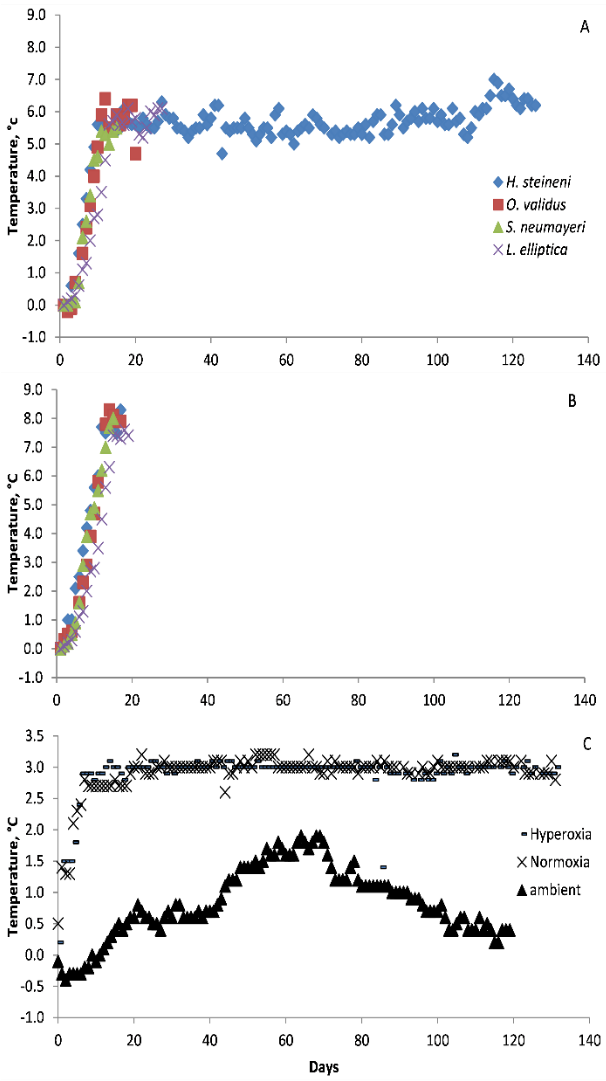

Figure S1. Example temperatures during ramping and incubation experiments at, A 6.0 °C and B 8.0 °C for 4 species *Heterocucumis steineni*, *Odontaster validus*, *Sterechinus neumeyeri* and *Laternula elliptica*, C for the 1st temperature-oxygen experiment.

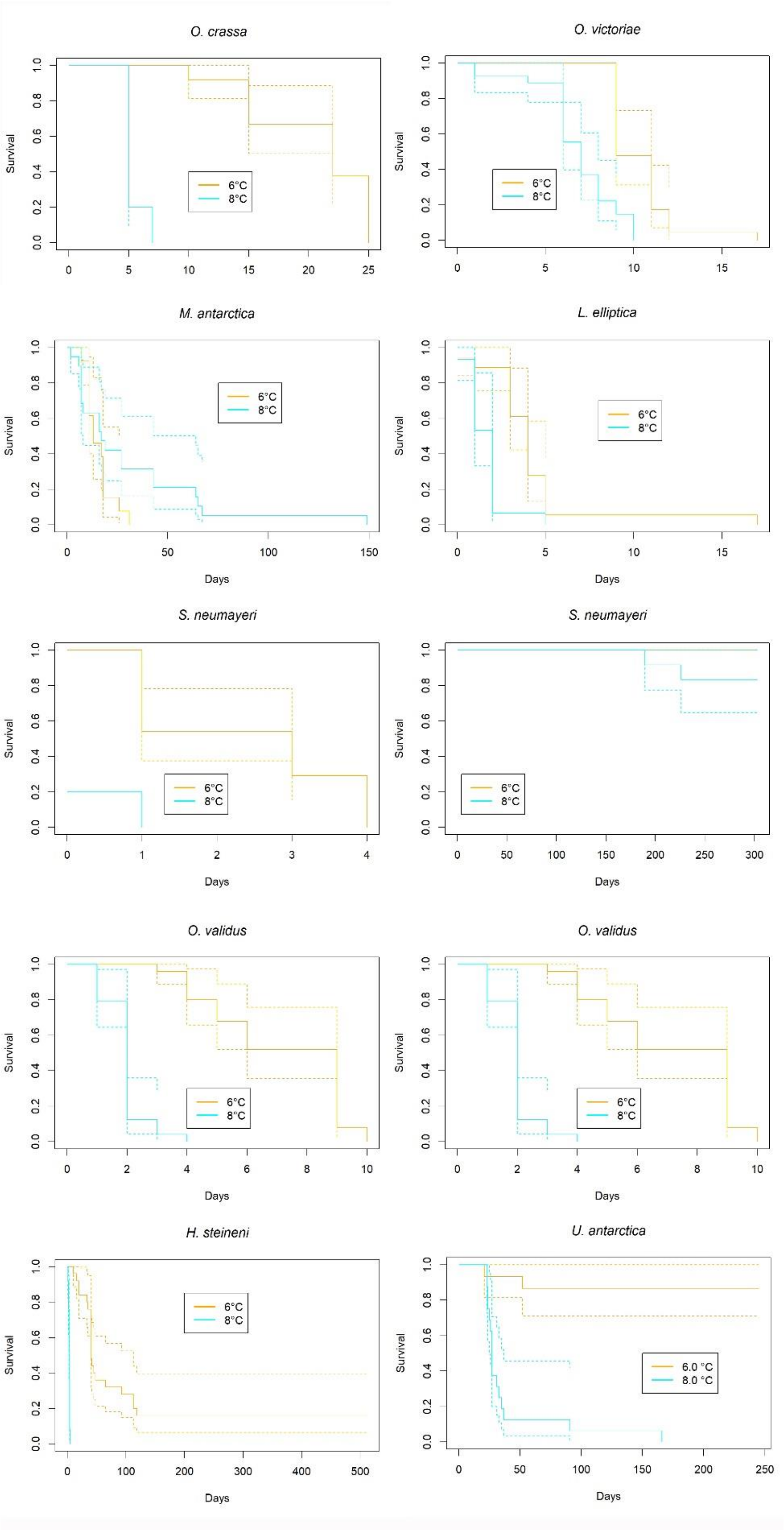

Fig S2. Kaplan Meier fits for survival of *Ophiura crassa, Ophionotus victoriae, Margarella antarctica, Laternula elliptica, Sterechnius neumayeri, Odontaster Validus, Hetercucumis steineni and Urticinopsis antarctica.*

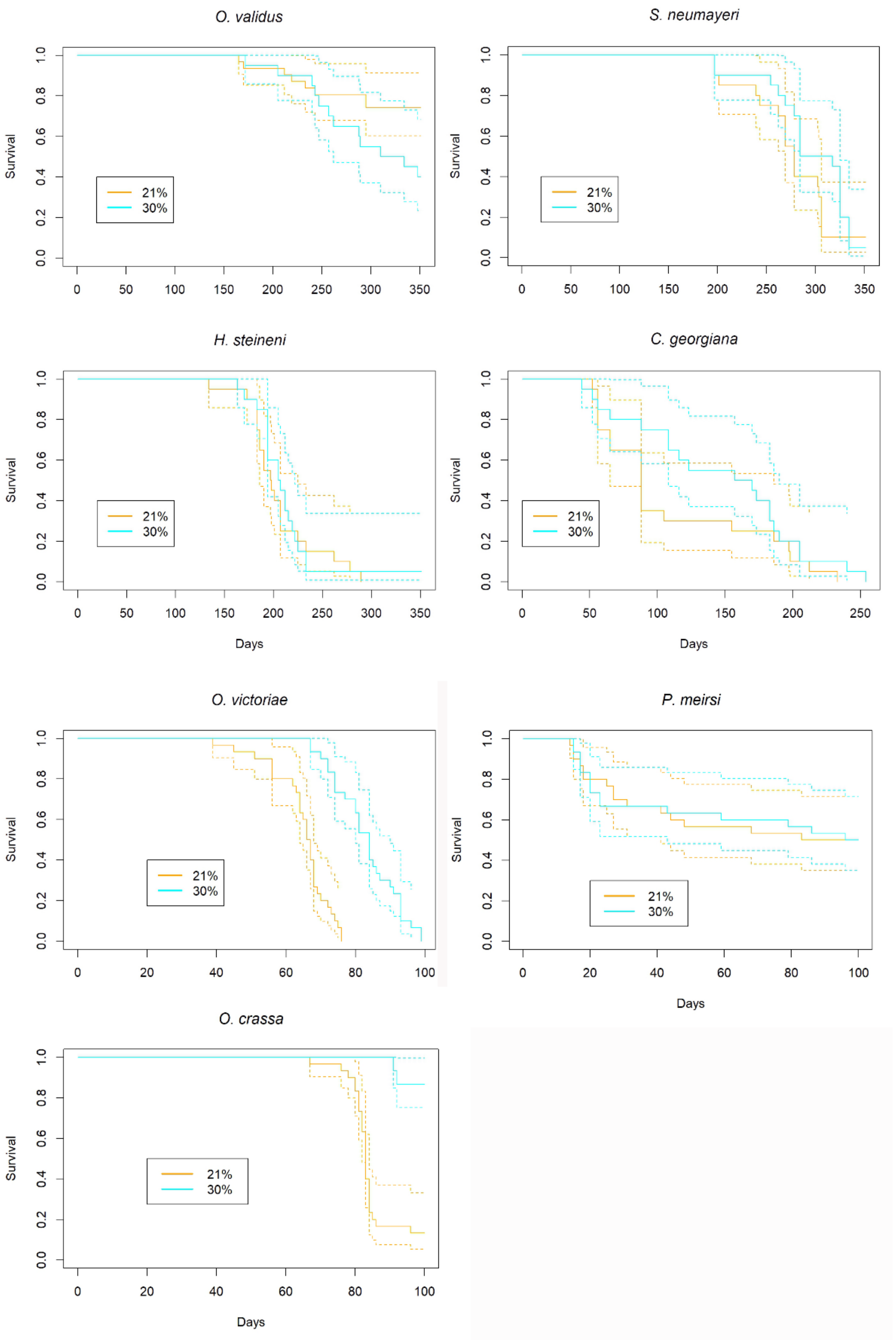

Fig S3. Kaplan-meier fits for survival of *Odontaster validus, Sterechinus neumayeri, Heterocucumis steineni, Cucumaria georgiana, Ophionotus victoriae, Paraceradocus miersi* and *Ophiura crassa* incubated at normoxia (21%) and hyperoxia (30%). See table 1 and Fig. 1 for incubation temperatures.

Table S1. Median and interquartile range from Kaplan Meier fits in days (± Inter-Quartile Range). Nominal temperatures of 6.0 and 8.0 °C are listed that had a typical SE of ± 0.04 °C (Fig. 1).

Species	Temp/ °C	Median	IQR	Mantel-Haenszel Chi^2	P
O. crassa	6.0	22	22-na		
	8.0	5	5-5	41.4	<0.01
O. victoriae	6.0	9	9-11		
	8.0	7	6-8	47.8	<0.01
M. antarctica	6.0	13	11-na		
	8.0	17	8-64	2.4	0.1
L. elliptica	6.0	4	3-5		
	8.0	2	1-2	13.4	<0.01
S. neumayeri	6.0	na	na		
	8.0	na	Na	3.7	0.06
O. validus	6.0	na	na	-	-
H. steineni	6.0	41	41-113		
	8.0	4	3-4	52.1	<0.01
U. antarctica	6.0	na	Na		
	8.0	27	25-37	25.6	<0.01

Table S2. Wet mass of incubated individuals, mean, standard error and sample size. Within each species, there were no significant differences in wet mass between treatments.

Species	accl Temp	tank	replicate	Mean	Sem	N
Urticinopsis antarctica	0	1		12.2	0.33	10
	0	2		11.2	0.91	9
	6	1		12.5	0.35	7
	6	2		13.0	0.34	6
Heterocucumis steineni	0	1	1	13.9	6.8	11
	0	2	1	12.9	3.4	11
	0	3	1	22.1	2.6	10
	0	3	2	33.5	4.6	8
	6	1	1	0.6	0.2	4
	6	2	1	22.7	2.3	10
	6	2	2	24.3	5.4	10
	6	3	1	27.1	4.4	10
	6	3	2	17.8	2.6	10
Odontaster validus	0	1	1	14.4	1.8	7
	0	1	2	12.1	2.7	4
	0	2	1	24.8	1.9	3
	6	1	1	7.9	0.9	10
	6	2	1	11.3	1.5	9
Sterechinus neumayeri	0	1		19.6	6.0	8
	0	2		21.6	3.4	8
	0	3		12.6	3.1	5
	6	1		25.8	2.0	9
	6	2		23.4	3.0	12
	8	1		19.6	3.2	10

Table S3. Median and interquartile range from Kaplan Meier fits for *Odontaster Validus, Sterechinus neumayeri, Heterocucumis steineni, Cucumaria georgiana, Ophionotus victoriae, Paraceradocus meirsi and Ophiura crassa* incubated at either normoxia (21%) and hyperoxia (30%).

Species	Temp	Oxygen	Median	IQR	Mantel-Haenszel ChiSq	P
O. validus	8.0 (10.0)	21	na	na		
	8.0 (10.0)	30	322	262-na	9.3	0.01
S. neumayeri	8.0 (10.0)	21	278	269-na		
	8.0 (10.0)	30	300	284-na	1.9	0.2
H. steineni	8.0 (10.0)	21	198	186-225		

	8.0 (10.0)	30	206	194-222	0.2	0.6
C. georgiana	8.0	21	88	65-186		
	8.0 (10.0)	30	164	108-190	1.8	0.2
O. victoriae	3.0	21	66.5	64-68		
	3.0	30	84	80-90	47.8	<0.01
P. meirsi	3.0	21	83	41-na		
	3.0	30	96	43-na	0	1
O. crassa	3.0	21	82	82-na		
	3.0	30	na	na	41.4	<0.01
